# First biochemical characterization of a novel ribonuclease from wild mushroom *Amanita hemibapha*

**DOI:** 10.1186/2193-1801-1-79

**Published:** 2012-12-27

**Authors:** Malota Sekete, Duanzheng Ma, Bo Wang, Hexiang Wang, Tzibun Ng

**Affiliations:** 1 State Key Laboratory for Agrobiotechnology and Department of Microbiology, China Agricultural University, Beijing, 100193 China; 2 Soil and Fertilizer Institute, Sichuan Academy of Agricultural Sciences, Sichuan, 610066 China; 3 School of Biomedical Sciences, Faculty of Medicine, The Chinese University of Hong Kong, Shatin, New Territories, Shatin, Hong Kong, China

**Keywords:** Ribonuclease, Mushroom, Purification

## Abstract

A 45-kDa ribonuclease (RNase) was purified from dried fruiting bodies of the wild mushroom *Amanita hemibapha*. It was adsorbed on DEAE-cellulose, S-sepharose, and finally purified on Superdex 75. The RNase exhibited maximal RNase activity at pH 5 and in a temperature range between 60-70°C. It demonstrated no ribonucleolytic activity toward four polyhomoribonucleotides. The amino acid sequence analysis (GDDETFWEHEWAK) showed this RNase was a ribonuclease T2-like RNase. It exhibited strong inhibitory activity against HIV-1 reverse transcriptase (HIV-1 RT) with an IC_50_ of 17 μM.

## Introduction

Ribonucleases (RNases) exist in a wide range of life forms from prokaryotes to eukaryotes (Fang and Ng. [2010]). RNases from different mushrooms also differ in biochemical properties such as molecular weight, carbohydrate content, and N-terminal sequence among others (Wang and Ng. [2006]). RNases isolated from different tissues may have different structures (Hofsteenge et al. [1989]; Iwama et al. [1993]; Sasso et al. [1991]) and it has long been claimed that wild mushrooms are beneficial to health in manifestation of anti-tumor (Kobayashi et al. [2000]), antiviral and antifungal (Wang and Ng. [2000]), immunomodulatory (Matousek et al. [1995]; Fang and Ng. [2010]) and immunosuppressive activities (Wang and Ng. [2000]; Ngai et al. [2003]). In this manner, their potential clinical importance may also one day find application in the treatment of chronic diseases such as cancer and HIV- 1 infection.

Ribonucleases are capable of offering protective measures to various organisms due to their host defense mechanisms (Wong et al. [2010]). Ribonucleases isolated from roots of *Panax ginseng* (Chinese ginseng), *P. notoginseng* (sanchi ginseng), and *P. quinquefolius* (American ginseng) have antifungal properties (Wang and Ng. [2000]). RNases of both Chinese and American ginseng are homodimeric and demonstrate HIV-1 reverse transcriptase inhibitory activity (Wang and Ng, [2000]).

RNases play a key role in RNA metabolism. They are involved in host defense and physiological cell death pathways. RNases possess therapeutic potentials for cancer treatment, as RNA damage caused by RNases could be an important alternative to standard DNA-damaging chemotherapeutics. (Makarov and Ilinskaya. [2003]). Four members of the RNase A superfamily : Onconase from oocytes of *Rana pipiens*, BS-RNase from bull semen, and two closely related sialic acid-binding lectins from oocytes of *Rana catesbeiana* and *Rana japonica* are endowed with antitumor activity and show cytotoxicity toward several tumor cell lines (Notomista et al. [2000]).

In the present study, a ribonuclease was isolated from the fryiting bodies of *Amanita hemibapha* for determination of biochemical characteristics and comparison with previously reported ribonucleases.

## Materials and Methods

Dried fruiting bodies of the mushroom *Amanita hemibapha* from Sichuan China were homogenized in 0.15 M NaCl solution using a Waring blender, and then stored at 4°C overnight before centrifugation (10000 g, 15 min). Ammonium sulfate precipitation was carried out by adding (NH_4_)_2_SO_4_ to the supernatant to 80% saturation to precipitate proteins. After centrifugation (10000 g, 15 min), the precipitated proteins were dissolved in distilled water and dialyzed to remove (NH_4_)_2_SO_4_. NaAc-HAc buffer (pH 5.6, 1 M) was added to the solution, until the concentration of NaAc reached 10 mM. The supernatant was subjected to ion exchange chromatography on a column of DEAE-cellulose (Sigma) in 10 mM NaAc-HAc buffer (pH 5.6). After elution of unadsorbed proteins (fraction D1) with the same buffer, adsorbed proteins were desorbed sequentially with 50 mM NaCl, 150 mM NaCl, and 1 M NaCl to yield fractions D2, D3, and D4, respectively. Fraction D3 with RNase activity was dialyzed and subsequently chromatographed on a 2.5×10 cm of S-Sepharose (Sigma) in 10 mM NaAc-HAc buffer (pH 3.6). After removal of unadsorbed proteins (fraction S1), adsorbed proteins were eluted with a linear concentration gradient (0–500 mM) of NaCl and 1 M NaCl in 10 mM NaAc-HAc buffer (pH 3.6) to yield fraction S2 and S3. The peak (S3) with RNase activity was then further purified on a Superdex 75 HR 10/30 column (GE health) in 0.15 M NH_4_HCO_3_ buffer (pH 8.5). The first peak (SU1) obtained represented purified RNase.

### Assay for activity of ribonuclease

Activity of *A. hemibapha* RNase toward yeast tRNA (Sigma) was assayed by measuring the production of acid-soluble, UV-absorbing species with a modification of the method of (Wang and Ng [2003a]). The RNase was incubated with 100 μg of tRNA in 150 μl 100 mM MES buffer (pH 4.6) at 37°C for 15 min. The reaction was terminated by addition of 350 μl of 3.7% perchloric acid. The sample was centrifuged at 15,000 g for 5 min. The absorbance of the resulting supernatant, after suitable dilution, was measured at 260 nm. One unit of enzymatic activity is defined as the amount of enzyme that produces an absorbance increase at 260 nm of one per minute in the acid-soluble fraction per milliliter of reaction mixture under the specified conditions. The optimal pH and temperature were determined following the same method as described using buffer with different pH values as the reaction buffer and different temperatures instead of 37°C.

### Molecular mass determination by sodium dodecyl sulfate polyacrylamide gel electrophoresis (SDS-PAGE) and by FPLC-gel filtration

SDS-PAGE was conducted in accordance with the procedure of ([Laemmli and Favre 1973]) using a 12% resolving gel and a 5% stacking gel. At the end of electrophoresis, the gel was stained with Coomassie brilliant blue. FPLC-gel filtration was carried out using a Superdex 75 HR 10/30 column that had previously been calibrated with molecular-mass standards using an AKTA Purifier (GE Healthcare).

### Analysis of partial amino acid sequence

The single band of the purified RNase from SDS-PAGE was cut out and sent to National Center of Biomedical Analysis (Beijing) for analysis of amino acid sequence by Q-TOF.

### Activity of *A. hemibapha* RNase toward polyhomoribonucleotides

The ribonucleolytic activity of *A. hemibapha* RNase toward polyhomoribonucleotides was determined with a modification of the method of ([Wang and Ng 2001]). Incubation of *A. hemibapha* RNase with 100 μg of poly A, poly C, poly G or poly U in 250 μl of 100 mM sodium acetate (pH 5.0) was carried out at 37°C for 1 h, prior to addition of 250 μl of ice-cold 1.2 N perchloric acid containing 20 mM lanthanum nitrate to terminate the reaction. After 15 min on ice, the sample was centrifuged at 15,000 ×g for 15 min at 4°C. The absorbance of the supernatant, after appropriate dilution, was read at 260 nm (for poly A, poly G and poly U) or at 280 nm (for poly C).

### Assay for ability to inhibit human immunodeficiency virus reverse transcriptase (HIV-1 RT)

The assay for ability of *A. hemibapha* RNase to inhibit HIV-1 RT activity was carried out as detailed by (Collins et al. [1997]) using a non-radioactive reverse transcriptase ELISA kit. The assay was executed following instructions supplied with the assay kit from Boehringer– Mannheim (Germany). The assay uses the ability to synthesize DNA with reverse transcriptase, starting from the template/primer hybrid poly(A) oligo(dT)_15._ The digoxigenin- and biotin-labeled nucleotides in an optimized ratio are incorporated into one of the same DNA molecule, which is freshly synthesized by the reverse transcriptase (RT). The detection and quantification of synthesized DNA as a parameter for RT activity follow an ELISA protocol. Biotin labeled DNA binds to the surface of microtiter plate modules that have been pre pre-coated with streptavidin. In the next step, an antibody to digoxigenin, conjugated to peroxidase, binds to the digoxigenin-labeled DNA. In the final step, the peroxidase substrate is added. The peroxidase enzymes catalyze the cleavage of the substrate, producing a colored reaction product. The absorbance of the samples at 405 nm can be determined using microtiter plate (ELISA) reader and is directly correlated to the level of RT activity. A fixed amount (4–6 ng) of recombinant HIV-1 reverse transcriptase was used. The inhibitory activity of the isolated ribonuclease was calculated as percent inhibition as compared to a control without the protein (Collins et al. [1997]).

## Results

**Figure 1:**
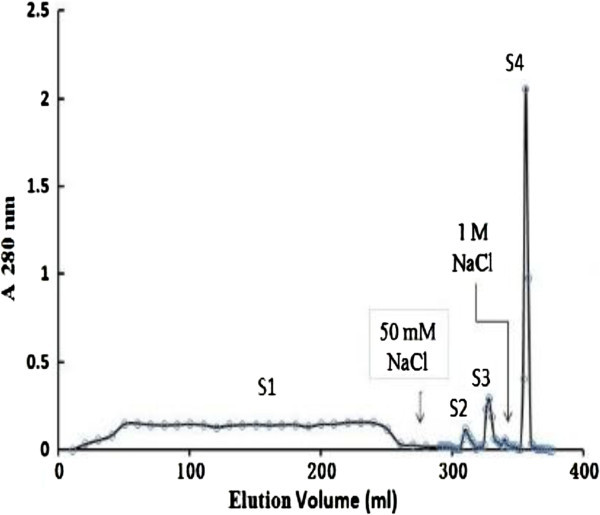
**Cation exchange chromatography of D3 on a S-Sepharose column which was first eluted with 10 mmol/L HAc-NaAc buffer (pH 3.6) and then stepwise with 50 mM and 1 M NaCl in the buffer.** RNase was detected only in fraction S3.

**Figure 2:**
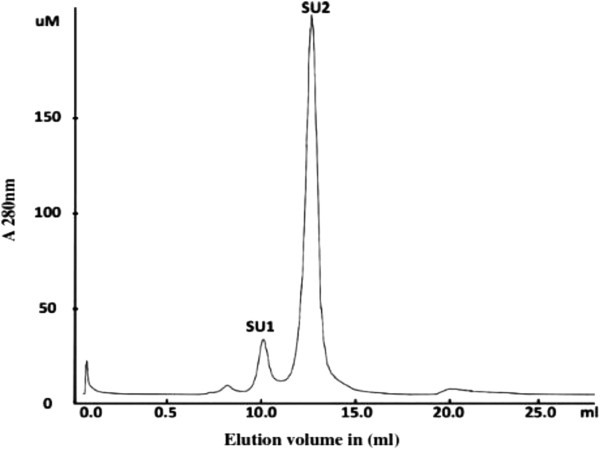
**FPLC-gel filtration of fraction S3 on a Superdex 75 HR10/30 column.** Buffer: 0.15 M NH_4_HCO_3_ (pH 8.5). Flow rate: 0.4 ml/min. Fraction size: 0.8 ml. RNase activity was confined to fraction SU1.

**Figure 3:**
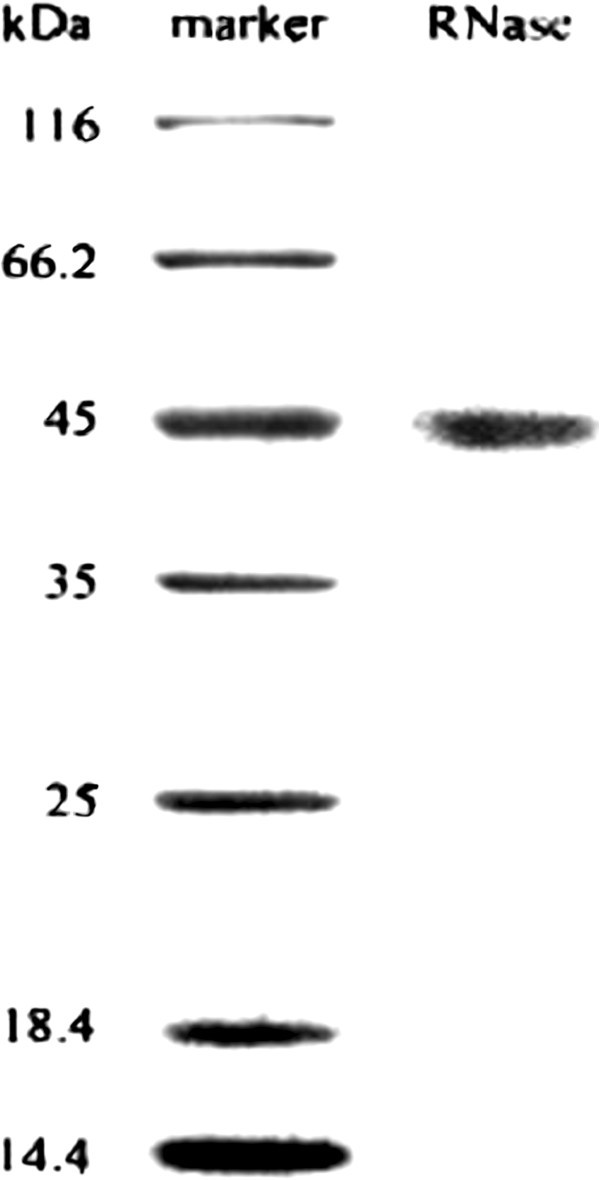
**SDS-PAGE results.** Left lane: molecular weight markers from Fermentas Life Sciences. Right lane: purified *A. hemibapha* RNase represented by fraction SU1 from Superdex 75 column.

Ion exchange chromatography of the crude sample on a DEAE-cellulose column yielded four fractions: D1 eluted with the starting buffer, and D2, D3, and D4 eluted sequentially with 50 mM NaCl, 150 mM NaCl, and 1 M NaCl in the buffer. Ribonuclease activity was detected only in fraction D3. D3 was separated on a S-Sepharose column into a broad unadsorbed fraction S1 eluted with the starting buffer, and two smaller fractions S2 and S3 eluted with 50 mM NaCl in the buffer, and the fraction S4 eluted with 1 M NaCl in the buffer (Figure 1). S3 was separated on Superdex 75 into a smaller peak (SU1) with activity and a large peak (SU2) without ribonuclease activity (Figure 2). The smaller fraction SU1 represented purified ribonuclease and exhibited a single band with a molecular mass of 45 kDa in SDS-PAGE (Table 1, Figure 3).

**Table 1:** **Yields and RNase activities of various chromatographic fractions**

Column	Fractions	Yield (mg)	Specific activity (U/mg)	Recovery of RNase activity (%)	Purification fold
	**Ammonium sulfate precipitate**	**540**	**14.24**	**100**	**1**
DEAE-cellulose	D1	12.85	15.38	<3	-
	D2	23.7	8.15	<3	-
	**D3**	**82.36**	**53.21**	**56.99**	**3.74**
	D4	255.7	2.79	<10	-
S-sepharose	D3S1	37.5	-	-	-
	D3S2	0.24	-	-	-
	**D3S3**	**6.78**	**186.07**	**16.41**	**13.07**
	D3S4	34	-	-	-
Superdex 75	**D3S3SU1**	**0.06**	**6746.67**	**5.29**	**473.78**
	D3S3SU2	0.697	-	-	-

The sequence of one peptide from T-TOF analysis was GDDETFWEHEWAK, which showed high homology (up to 100%) with Ribonuclease Trv and Ribonuclease T2 of many microorganisms by BLAST search. These ribonuclease sequences are compared in Table 2.

**Table 2:** **Partial sequence of*A. hemibapha*RNase in comparison with other reported RNases**

Protein	Sequence	Accession
*A. hemibapha* RNase of this study	GDDETFWEHEWAK	
ribonuclease Trv [*Metarhizium anisopliae*]	125 GDDETFWEHEWAK 137	EFZ03379.1
ribonuclease T2 [*Puccinia graminis*]	144 GNDETFWEHEWAK 156	XP_003335329.1
ribonuclease T2 family, putative [*Trichophyton verrucosum*]	125 GDDETFWEHEWNK 137	XP_003023932.1
ribonuclease T2 [*Morchella esculenta*]	124 GDDESFWEHEWSK 136	BAK32788.1
ribonuclease M [*Cordyceps militaris*]	117 GDDESFWEHEWGK 129	EGX96697.1
ribonuclease T2 precursor [*Aspergillus terreus*]	123 GDDESFWEHEWNK 135	XP_001214320.1
ribonuclease T2 [*Aspergillus fumigates*]	128 GDDESFWEHEWNK 140	XP_753027.1
S-like RNase [*Volvox carteri*]	120 DETFWEHEWSK 130	XP_002947011.1

Different amino acid residues are underlined. Data are taken from NCBI BLAST.

**Figure 4:**
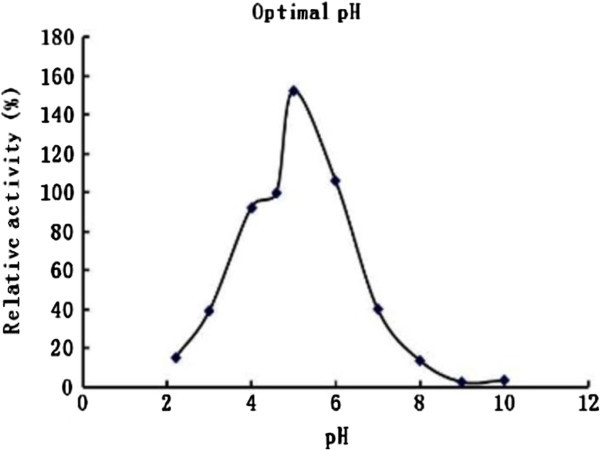
**Optimal pH for*A. hemibapha*RNase.**

**Figure 5:**
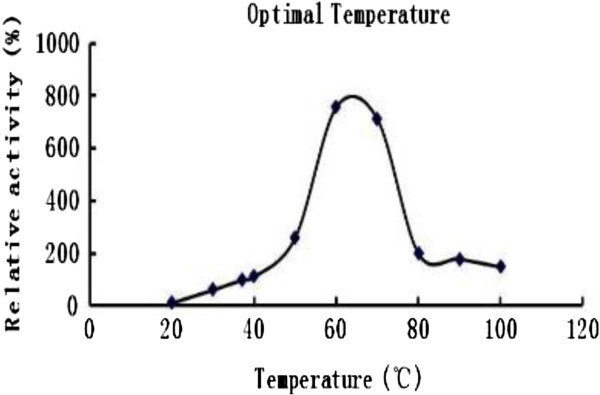
**Optimal temperature for*A. hemibapha*RNase.**

The activity of ribonuclease reached a maximum at pH 5.0, and dropped precipitously when the pH was lowered below 4 or raised above 7 (Figure 4). The optimal temperature was 60~70°C (Figure 5). *A. hemibapha* RNase inhibited HIV-1 reverse transcriptase with an IC_50_ of 17 μM. A comparison of *A. hemibapha* RNase with previously published mushroom RNases is shown as a supplement in Table 3.

**Table 3:** **Comparison of characteristics of various mushroom ribonucleases**

	Mol mass (kDa)	Optimum pH	Optimum temperature	HIV-1 RT inhibitory activity (IC_50_in μM)	Antiproliferative activity (IC_50_in μM)		
					L1210 cells	Hep G2 cells	MCF-7 cells
*A. hemibapha*	45	5.0	60~70	17	ND	UD	UD
*Boletus griseus*	29	3.5	60-70	ND	ND	ND	ND
*Clitocybe maxima*	17.5	6.5, 7.0	70	ND	ND	ND	ND
*Dictyophora indusiata*	28	4-4.5	60	ND	ND	ND	ND
*Ganoderma lucidum*	42	4	60	ND	ND	ND	ND
*Hypsizigus marmoreus*	18	5	70	ND	60	ND	ND
*Lyophyllum shimeiji*	14.5	6	70	7.2	ND	10	6.2
*Pleurotus djamor*	15	4.6	60	ND	ND	3.9	3.4
*Pleurotus eryngii*	16	6.5	70	ND	ND	ND	ND
*Pleurotus ostreatus* (Nomura et al.)	12.4	8.0	ND	ND	ND	ND	ND
*Pleurotus ostreatus* (Ye et al.)	12	7.0	40	ND	ND	ND	ND
*Pleurotus pulmonarius*	14.4	7	55	ND	ND	ND	ND
*Pleurotus sajor-caju*	12	5.5	<60	ND	0.1	0.22	ND
*Pleurotus tuber-regium*	29	6.5	ND	ND	ND	ND	ND
*Russula delica*	14	5	60	UD	ND	8.6	7.2
*Russulus virescens*	28	4.5	60	ND	ND	ND	ND
*Thelephora ganbajun*	30	6-7	40	0.3	ND	ND	ND
*Volvariella volvacea*	42.5	6.5,-7.5	ND	ND	ND	ND	ND

## Discussion

A 45-kDa ribonuclease (RNase) purified from dried fruiting bodies of the wild mushroom *Amanita hemibapha* is reported herein. Its molecular size of 45 kDa falls outside the range exhibited by all mushroom ribonucleases (9-45 kDa) reported so far, being greater than that of straw mushroom (42 kDa) (Wang and Ng. [1999]). RNases from *Boletus griseus* (Wang and Ng. [2006]), *Clitocybe maxima* (Wang and Ng. [2004a]), *Dictyophora indusiata* (Wang and Ng. [2003a]), *Hypsizigus marmoreus* (Guan et al. [2007]), *Lyophyllum shimeiji* (Zhang et al. [2010]), *Pleurotus djamor* (Wu et al. [2010]), *Pleurotus eryngii* (Ng and Wang, [2004]), *Pleurotus ostreatus* (Ye and Ng. [2003]), *Pleurotus pulmonarius* (Ye and Ng, [2002]), *Pleurotus sajor-caju* (Ngai and Ng. [2004]), *Pleurotus tuber-regium* (Wang and Ng. [2001]), *Russula delica* (Zhao et al. [2010]), *Russulus virescens* (Wang and Ng [2003b]) and *Thelephora ganbajun* (Wang and Ng [2004b]), are all smaller than 32 kDa while those of *Ganoderma lucidum* (Wang et al. [2004]) and *Volvariella volvacea* (Wang and Ng. [1999]) are higher than 30 kDa but less than 45 kDa. *A. hemibapha* RNase demonstrated no ribonucleolytic activity toward four polyhomoribonucleotides. Most reported RNases such as *P. tuber-regium* (Wang and Ng. [2001]) and *P. ostreatus* (Nomura et al. [1994]) RNases are specific for poly G. *L. edodes* RNase exhibits preference for polyA (Kobayashi et al. [1992]). Others like an ubiquitin-like peptide from mushroom *Cantharellus cibarius* (Wang et al. [2003]) showed ribonuclease activity against various polyhomoribonucleotides.

The optimum pH was 5 and the optimal temperature was 60~70°C. Its optimum pH is very different from that of *Russulus virescens* ribonuclease (optimum pH of 4.5) (Wang and Ng [2003b]) and of *Ganoderma lucidum* ribonuclease (optimum pH of 4.0) (Wang et al. [2004]). The temperature dependence curve for the activity of *A. hemibapha* ribonuclease indicates that it is a fairly thermostable enzyme. It retains more than half of its maximal activity at 80°C and is totally inactivated only at 100°C. The partial sequence of *A. hemibapha* RNase reveals 100% similarity to ribonuclease Trv *Metarhizium anisopliae* and slight difference from ribonuclease T2 *Puccinia graminis*, ribonuclease T2 family, putative *Trichophyton verrucosum*, ribonuclease T2 *Morchella esculenta*, ribonuclease M *Cordyceps militaris*, ribonuclease T2 precursor *Aspergillus terreus*, ribonuclease T2 *Aspergillus fumigatus*, S-like RNase *Volvox carteri* as depicted in Table 2. But this RNase inhibited HIV-1 reverse transcriptase with an IC_50_ of 17 μM. This anti-HIV-1 reverse transcriptase activity has not been demonstrated for the majority of the previously isolated mushroom RNases.
